# Innate and adaptive immunity to human beta cell lines: implications for beta cell therapy

**DOI:** 10.1007/s00125-015-3779-1

**Published:** 2015-10-21

**Authors:** Cornelis R. van der Torren, Arnaud Zaldumbide, Dave L. Roelen, Gaby Duinkerken, Simone H. Brand-Schaaf, Mark Peakman, Paul Czernichow, Philippe Ravassard, Raphael Scharfmann, Bart O. Roep

**Affiliations:** Department of Immunohematology and Blood Transfusion, Leiden University Medical Center, E3-Q, P.O. Box 9600, 2300 RC Leiden, the Netherlands; Department of Molecular Cell Biology, Leiden University Medical Center, Leiden, the Netherlands; Department of Immunobiology, School of Medicine, King’s College London, London, UK; Endocells, Paris, France; CNRS UMR 7725, Université Pierre et Marie Curie (UPMC), Paris, France; Inserm U1016, Université Paris Descartes, Sorbonne Paris Cité, Paris, France

**Keywords:** Adaptive immunity, Beta cell, Innate immunity, Transplantation

## Abstract

**Aims/hypothesis:**

Genetically engineered human beta cell lines provide a novel source of human beta cells to study metabolism, pharmacology and beta cell replacement therapy. Since the immune system is essentially involved in beta cell destruction in type 1 diabetes and after beta cell transplantation, we investigated the interaction of human beta cell lines with the immune system to resolve their potential for immune intervention protocol studies.

**Methods:**

Human pancreatic beta cell lines (EndoC-βH1 and ECi50) generated by targeted oncogenesis in fetal pancreas were assessed for viability after innate and adaptive immune challenges. Beta cell lines were pre-conditioned with T helper type 1 (Th1) cytokines or high glucose to mimic inflammatory and hyperglycaemia-stressed conditions. Beta cells were then co-cultured with auto- and alloreactive cytotoxic T cells (CTL), natural killer (NK) cells, supernatant fraction from activated autoreactive Th1 cells, or alloantibodies in the presence of complement or effector cells.

**Results:**

Low HLA expression protected human beta cell lines from adaptive immune destruction, but it was associated with direct killing by activated NK cells. Autoreactive Th1 cell inflammation, rather than glucose stress, induced increased beta cell apoptosis and upregulation of HLA, increasing beta cell vulnerability to killing by auto- and alloreactive CTL and alloreactive antibodies.

**Conclusions/interpretation:**

We demonstrate that genetically engineered human beta cell lines can be used in vitro to assess diverse immune responses that may be involved in the pathogenesis of type 1 diabetes in humans and beta cell transplantation, enabling preclinical evaluation of novel immune intervention strategies protecting beta cells from immune destruction.

**Electronic supplementary material:**

The online version of this article (doi:10.1007/s00125-015-3779-1) contains peer-reviewed but unedited supplementary material, which is available to authorised users.

## Introduction

Beta cell replacement by pancreas or islet transplantation is currently the only curative treatment for established type 1 diabetes. Insulin independence using current islet transplantation protocols is often temporary despite aggressive immune suppression. Both innate and adaptive immune responses threaten transplanted beta cells and need to be controlled by immune suppression [1–3]. More effective and less toxic strategies are required to make beta cell transplantation affordable to more patients.

Knowledge of interactions of human beta cells with the immune system has been largely derived from studies on isolated islets from pancreas donors. Access to such preparations for scientific purposes is limited; furthermore, variations between islet preparations and their composition, including a range of other cell types, hinder beta cell-specific studies. Human genetically engineered beta cell lines provide a novel tool to study functional human beta cells in standardised assays [4]. Thus, beta cell lines may help to identify immune responses relevant to human type 1 diabetes and beta cell transplantation.

We investigated innate and adaptive immune responses potentially harmful to beta cells in the pathogenesis of type 1 diabetes and beta cell transplantation on genetically engineered human beta cell lines to assess their potential for preclinical evaluation of novel immune intervention strategies.

## Methods

Two human fetal beta cell lines with similar function (EndoC-βH1 and ECi50; Endocells, Paris, France) were generated and maintained as previously reported [4]. To mimic inflammation or hyperglycaemia, beta cell lines were preincubated overnight with IFNγ (1,000 U/ml; R&D Systems, Abingdon, UK) or glucose 20 mmol/l. Introduction of EF1α promoter-driven HLA-A*02:01 into beta cell line EndoC-βH1 was achieved by lentiviral transduction [5]. HLA genotyping was carried out at the Eurotransplant Reference Laboratory, Leiden University Medical Center, Leiden, the Netherlands.

Informed consent and approval of the institutional review board was obtained for the generation of human cell lines and antibodies and was carried out in accordance with the 2008 revised principles of the Declaration of Helsinki.

Peripheral blood mononuclear cells (PBMC) were separated from full blood or buffy coats (for natural killer [NK] cells and lymphocytes) by Ficoll-Hypaque density gradient. Peripheral blood lymphocytes (PBL) were separated by CD14 depletion of PBMC with CD14 MicroBeads (Miltenyi Biotec, Auburn, CA, USA). NK cells were purified from PBMC using the human NK Cell Isolation Kit (Miltenyi Biotech, Leiden, the Netherlands), cultured and activated with IL-15 as described [6]. Details about generation and maintenance of specific T cell clones, immortalised human primary tubular epithelial cells (PTEC), HeLa, Epstein–Barr virus-transformed B lymphocytes, mesenchymal stromal cells (MSC) and human monoclonal antibodies recognising HLA have been previously published [7–11].

Beta cell-specific T helper (Th) cell supernatant fraction was harvested from 3 day cultures of autoreactive Th1 clone 1c6 incubated with PBMC and preincubated with or without antigen [12]. Supernatant fraction was stored at −80°C until use.

Cellular cytotoxicity was assessed by chromium release of ^51^Cr-labelled beta cell lines. Complement-dependent cytotoxicity was measured by flow cytometry of beta cell lines after incubation with human HLA-specific antibodies and rabbit complement. Cytokine-driven beta cell death was measured by propidium iodide staining and flow cytometry after 48 h culture in Th1 cell supernatant fraction or 50 U/ml IL-1β, 1,000 U/ml IFNγ and 1,000 U/ml TNF-supplemented medium. Cell surface antigen expression was assessed by flow cytometry.

Experiments were not blinded. Experiments were excluded if positive controls did not respond or with responding negative controls. Mycoplasma infection was excluded for all cell lines at regular intervals.

Data are represented as mean and SD unless stated otherwise. Statistics represent linear regression for titrated experiments and Student’s *t* test for binary outcomes. GraphPad Prism 6.0 (GraphPad Software, La Jolla, CA, USA) was used to create graphs and perform analysis. Further details are given in the electronic supplementary material (ESM methods).

## Results

### Cytokine-mediated effects on beta cells

**Fig. 1:**
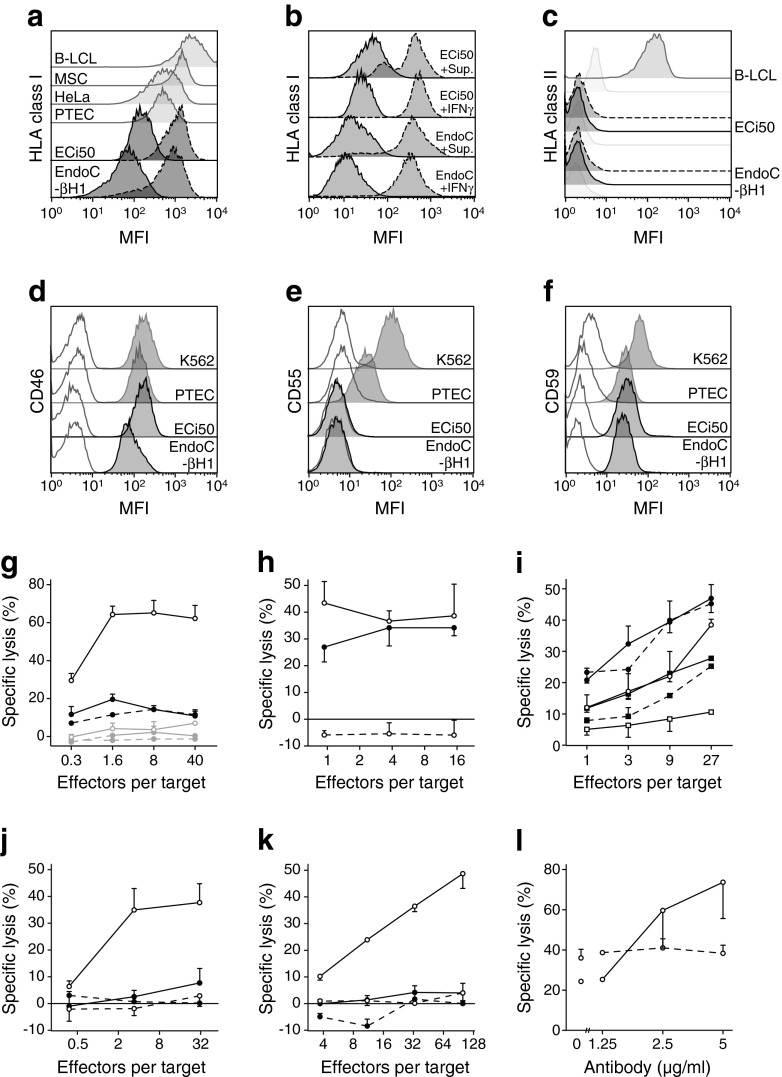
(**a**–**c**) HLA class I and class II expression was measured in beta cell lines EndoC-βH1 and ECi50 and compared with other cell lines. HLA expression was stimulated (dashed line) through incubation with supernatant fraction (Sup.) of a beta cell-specific Th1 cell response or inflammatory cytokine IFNγ; isotype controls are shown in light grey. (**d**–**f**) Expression of complement inhibitory receptors CD46, CD55 and CD59 on beta cell lines compared with other cell lines. (**g**–**i**) Cellular cytotoxic responses to beta cell lines tested in chromium release assays. (**g**) Alloreactive (*HLA-A2*-specific) CTLs vs beta cells expressing *HLA-A2* (black lines and symbols) or not expressing *HLA-A2* (grey lines and symbols). Unconditioned beta cells (solid black/grey lines and symbols) were compared with HLA upregulated beta cells by IFNγ (solid black/grey lines and white symbols) and glucose-stimulated beta cells (dashed lines and black/grey symbols). (**h**) Autoreactive PPI-specific CTLs vs HLA-A*02:01-transduced beta cells presenting peptide from endogenously produced insulin (black symbols) or presenting exogenous loaded peptide (white symbols), and mock transduced cells, in the presence of exogenous peptide (dashed line). (**i**) Activated NK cells vs EndoC-βH1 (circles) and ECi50 (squares). Unconditioned beta cells (black lines and symbols) were compared with HLA upregulated cells (solid lines and white symbols) and glucose-stimulated cells (dashed lines and black symbols). (**j**–**l**) Alloreactive antibodies, specific (solid lines) or non-specific (dashed lines), for EndoC-βH1 HLA-induced lysis by (**j**) NK cells and (**k**) PBL, and (**l**) through complement-dependent cytotoxicity without (black symbols) or after (white symbols) HLA upregulation by IFNγ. Data are presented as mean and SD; panels show representative experiments. *x*-axes are plotted on logarithmic scales

Two human beta cell lines (EndoC-βH1 and ECi50) were selected for immunological analysis. Cells were genotyped as HLA-A*33:03, A*68:01 (EndoC-βH1) and HLA-A*02:02, A*68:01 (ECi50). HLA class I expression on EndoC-βH1 was slightly lower than on ECi50 (geo-mean fluorescence intensity [MFI] 21 vs 59), and much lower than HLA expression on various non-beta cell lines (B-lymphoblastoid cell lines [B-LCL]: MFI 2146; MSC: MFI 1299; PTEC: MFI 479; HeLa: MFI 481). HLA class I expression could be upregulated by IFNγ (sixfold on ECi50, ninefold on EndoC-βH1), while HLA class II expression remained absent (Fig. 1a, c).

To assess the influence of autoimmune inflammation on beta cell lines, cells were cultured in 3 day culture supernatant fraction of activated islet autoreactive Th1 cells containing IL-1β (16 pg/ml), IL-13 (113 pg/ml), IL-17 (36 pg/ml), IFNγ (1,000 pg/ml) and TNF (18 pg/ml) for 48 h. Supernatant fraction of activated T cells increased HLA class I, but not class II, expression, similar to incubation with IFNγ (Fig. 1b).

Supernatant fraction of activated T cells increased beta cell death from 46 ± 5% to 70 ± 2% (*p* < 0.0001; *n* = 3) for EndoC-βH1, and from 36 ± 6% to 59 ± 5% (*p* < 0.0001; *n* = 3) for ECi50. Comparably, incubation with mixed cytokines (IFNγ 1,000 U/ml, TNF 1,000 U/ml and IL-1β 50 U/ml) increased beta cell death from 22 ± 6% to 40 ± 8% (*p* = 0.0003; *n* = 4) for EndoC-βH1 and from 22 ± 5% to 35 ± 8% (*p* = 0.0002; *n* = 4) for ECi50. This resembles the effect described on islets [2]. Individual cytokines did not induce apoptosis.

### Cell-mediated cytotoxicity

Destruction of beta cells by autoreactive cytotoxic T cells (CTL) is the hallmark of type 1 diabetes. We therefore investigated autoreactive preproinsulin (PPI)-specific CTL responses to endogenous expression of beta cell antigens by the cell lines. Since our effector T cell clones are *HLA-A2* (*02:01)-restricted and the beta cell lines were lacking *HLA-A2*, expression had to be introduced. Beta cell line EndoC-βH1 was transduced with HLA-A*02:01 under the elongation factor 1-alpha (EF1α) promotor. After passaging, the generated line contained 39% *HLA-A2*-positive cells and was stable for at least 12 passages. Expression of transduced HLA-A*02:01 was MFI 118 and was unaffected by IFNγ.

Overnight incubation of the *HLA-A2*-transduced beta cell line with PPI-specific cytotoxic T cells resulted in beta cell cytolysis up to 34 ± 3% (*p* < 0.0001 for intercept; *n* = 4) without adding exogenous PPI peptide epitope, corresponding to *HLA-A2* expressing cell frequency (Fig. 1h). Pulsing of the transduced beta cell with exogenous cytomegalovirus (CMV) peptide epitope (mimicking CMV infection) resulted in killing by CMV-specific CTLs with similar efficacy (data not shown).

Alloreactive CTLs can cause beta cell allograft rejection after transplantation. Thus, beta cells were tested against HLA-A*02:02-specific alloreactive CTLs. A beta cell line naturally expressing HLA-A*02:02 was killed (up to 66 ± 5%) in a 4 h cytotoxicity assay only if HLA was upregulated by IFNγ (*p* = 0.005 for intercept; *n* = 3). Hyperglycaemic (>25 mmol/l glucose) preincubation did not affect killing by alloreactive CTLs (Fig. 1g). Specific recognition of beta cell lines by alloreactive CTLs after HLA upregulation was verified by expression of the cytolytic degranulation marker CD107a on responding CTLs (data not shown).

Low HLA expression by the beta cell lines may render these cells susceptible to NK cell reactivity. Indeed, activated NK cells killed beta cell line EndoC-βH1, which expresses relatively less HLA more efficiently than ECi50 (up to 47 ± 4% and 28 ± 0%, respectively; *p* = 0.016 for slope; *n* = 2). HLA upregulation reduced killing to 38 ± 2% (*p* = 0.002 for intercept) for EndoC-βH1 and 11 ± 1% (*p* = 0.0003 for slope) for ECi50 (Fig. 1i). Hyperglycaemia did not influence NK cell killing of beta cell lines. Results were corroborated by a CD107a degranulation assay (data not shown).

### Antibody- and complement-mediated killing

Antibodies recognising HLA can lead to acute rejection of transplants through activation of immune cells or complement. Low HLA expression protected from antibody-dependent cellular cytotoxicity by PBL or purified NK cells. Yet, HLA upregulation increased killing through alloreactive antibodies (for EndoC-βH1 up to 38 ± 7% through NK cells [*p* = 0.002 for intercept; Fig. 1j]; and up to 49 ± 6% through PBL [*p* < 0.0001 for slope; Fig. 1k]). Complement inhibitory receptors generally prevent direct complement activation, and beta cell lines expressed CD59 and CD46, but not CD55 (Fig. 1d–f). Beta cell lines were thereby protected from killing by human serum complement.

To assess their killing potential, alloantibodies were titrated in standard clinical cross-match assays using rabbit complement. Specific alloreactive antibodies induced >80% complement-dependent cytotoxicity of beta cell lines upon upregulation of HLA by IFNγ, whereas alloantibodies directed to HLA not expressed by the human beta cell lines had no such effect (*p* = 0.006 for slope) (Fig. 1l).

## Discussion

We investigated immune responses to human beta cell lines that may be relevant for diabetes pathogenesis and beta cell transplantation, demonstrating the relevance of these beta cell lines for preclinical studies on immune intervention strategies (Table 1).

**Table 1:** Overview of results

Interaction with immune system	Conditioning of beta-cell lines		
	Resting	Inflammatory cytokines	Glucose challenge
HLA expression	Lower than other tissue cell lines	Increased Higher than basal expression of other tissue cell lines	Unchanged (i.e. low)
Autoreactive Th cell supernatant	Moderate apoptosis	NA	Moderate apoptosis
Autoreactive CTL recognition and killing	Proinsulin-specific killing	NA	Proinsulin-specific killing
Alloreactive CTL recognition and killing	Immune response Moderate killing	Strong immune response Effective killing	Immune response Moderate killing
NK cell recognition and killing	Recognition and differential killing	Decreased killing	Recognition and differential killing
ADCC	No killing with HLA antibodies	Concentration-dependent killing	ND
CDC	No killing with HLA antibodies	Concentration-dependent killing	ND

ADCC, antibody-dependent cellular cytotoxicity; CDC, complement-dependent cytotoxicity; NA, not applicable; ND, no data

Studies of type 1 diabetic pancreases suggest that autoreactive cytotoxic T cells are highly efficient killers of beta cells [13]. We confirm that autoreactive T cell clone 1E6 can efficiently kill the beta cell lines that were HLA compatible, which substantiates that these beta cell lines can process and present PPI_15–24_ epitope from endogenously produced PPI to the immune system. This establishes these cell lines as bona fide beta cells in terms of their susceptibility to diabetogenic autoimmune reactions.

Alloreactive responses may be detrimental for transplanted beta cells too. We show that beta cell lines become sensitive to killing by donor-specific alloreactive CTLs or alloantibodies if HLA is upregulated by inflammation. At the same time, low HLA expression left unstimulated beta cell lines vulnerable to activated NK cells. These data support clinical observations that suppressing early inflammation may be as important for transplant success as immunosuppression targeting adaptive immunity.

Whether normal human beta cells express equally low HLA remains unknown, since HLA expression by human beta cells purified from isolated islets is difficult to quantify. However, HLA class I is markedly upregulated in pathogenic conditions including insulitis in islets of type 1 diabetic patients [13]. We confirm that supernatant fraction of autoreactive T cells from a patient with type 1 diabetes responding to islet antigen can upregulate HLA on beta cell line cells. Moreover, these supernatant fractions increased beta cell death, similar to previously described inflammatory cytokines [2].

In conclusion, we demonstrate that genetically engineered human beta cell lines can be used in vitro to assess diverse immune responses that may be involved in the pathogenesis of type 1 diabetes in humans and in beta cell transplantation. This enables human preclinical evaluation of novel immune intervention strategies protecting beta cells.

## Electronic supplementary material

ESM Methods (PDF 57 kb)

## Abbreviations

B-LCL: B-lymphoblastoid cell lines
CMV: Cytomegalovirus
CTL: Cytotoxic T cells
EF1α: Elongation factor 1-alpha
MFI: Mean fluorescence intensity
MSC: Mesenchymal stromal cell
NK: Natural killer
PBL: Peripheral blood lymphocytes
PBMC: Peripheral blood mononuclear cells
PPI: Preproinsulin
PTEC: Primary tubular epithelial cell
Th1: T helper type 1
