# Supplementary material for: Innate and adaptive immunity to human beta cell lines: implications for beta cell therapy
Source: Diabetologia. 2015 Oct 21;59(1):170–5. doi: 10.1007/s00125-015-3779-1 (PMC4670455; doi:10.1007/s00125-015-3779-1)
Supplement: Supplementary file 1 — (PDF 57 kb) [file 125_2015_3779_MOESM1_ESM.pdf]

Online supplementary material:

Extended Methods Section

Cell lines and antibodies

Two human fetal beta cell lines (EndoC-βH1 and ECi50; Endocells, Paris, France) were used of which generation and functionality have been reported in detail before [1]. Cells were cultured in vitro in Matrigel/fibronectin–coated (100 μg/ml and 2 μg/ml, respectively; Sigma-Aldrich, Zwijndrecht, The Netherlands) in DMEM containing 1 g/L D-glucose and Glutamax (Gibco, Life Technologies, Bleiswijk, The Netherlands) supplemented with 2% BSA fraction V (Roche Diagnostics, Woerden, The Netherlands), 50 μM 2-mercaptoethanol, 10 mM nicotinamide (Calbiochem, Amsterdam, The Netherlands), 5.5 μg/ml transferrin (Sigma-Aldrich), 6.7 ng/ml selenite (Sigma-Aldrich),100 U/ml penicillin, and 100 μg/ml streptomycin (Gibco). Cells were harvested after 5 minutes incubation in 0.05% trypsin-EDTA 1X (Gibco,) for passaging and immune assays. To mimic inflammation or hyperglycemia, beta-cell lines were pre-incubated overnight with IFNγ (1000 U/ml; R&D systems, Abingdon, United Kingdom) or 20 mM glucose.

Peripheral blood mononuclear cells (PBMC) were separated from full blood (to collect T-cells) or buffy coats (for NK-cells & lymphocytes) by Ficoll-Hypaque density gradient. Peripheral blood lymphocytes were separated by CD14 depletion of PBMC with CD14-MicroBeads according to the manufacturer's protocol (Miltenyi Biotec, Auburn, CA, USA). NK cells were purified from PBMC by negative selection using the "Human NK cell Isolation Kit" (Miltenyi Biotech, Leiden, The Netherlands). NK cells were cultured in AIM-V medium with 2 mmol/L of glutamine (Invitrogen, Life Technologies) supplemented with 10% of pooled human AB serum (Sanquin, Amsterdam, The Netherlands), penicillin/streptomycin and could be activated by supplementing with 10 ng/mL of IL-15 (Peprotech, London, United Kingdom) for 1 to 4 weeks [2].

The generation of a preproinsulin specific T-cell clone 1E6 generation was described previously [3, 4]. Several cell lines were used for reference. Immortalized human PTEC (HK-2) were grown as

previously described [5]. HeLa cells and EBV transformed B-lymphocytes were cultured in IMDM with 5% fetal bovine serum (Lonza, Breda, The Netherlands). Human mesenchymal stromal cells were cultured as previously described [6].

Beta-cell specific Th-cell supernatant was generated from Th1 clones reactive to islet preparation (1c6) derived from diabetic patients [7]. T-cells were incubated in 1:5 ratio with peripheral blood mononuclear cells pre-incubated with or without antigen at 10 µg/ml in RPMI 1640 medium (Gibco) or Iscove's Modified Dulbecco's Medium (Lonza, Basel, Switserland), with 5.6 mM or 25 mM D-glucose and supplemented with 2 mMol/l glutamine (Gibco). After 3 days supernatant was harvested, aliquoted and stored at -80°C until use.

Human monoclonal antibodies recognizing HLA-A68 or HLA-B8 were selected from a panel described elsewhere [8]. In short, hybridomas were created by EBV transformation and cloning of B-lymphocytes of multiparous women fused with myeloma cell lines. The HLA-specificities of the produced human mAbs were validated using complement-dependent cytotoxicity test against peripheral blood mononuclear cells. For compatibility with autoreactive CTL clone 1E6, beta-cell line EndoC-βH1 was transduced with a lentiviral vector containing HLA-A02:01 under EF1α promotor at MOI=2. Third generation self-inactivating lentivirus vectors were produced as described previously [9]. HLA genotyping was performed by the Eurotransplant reference laboratory at the Leiden University Medical Center, Leiden, The Netherlands.

Informed consent and approval of institutional review board was obtained for generation of human cell lines and antibodies and have been carried out in accordance with the 2008 revised principles of the Declaration of Helsinki.

Cytotoxicity assays

Cellular cytotoxicity was assessed by chromium release (PerkinElmer, Waltham, MA, USA). Briefly, cells were harvested and labeled with ^{51}Cr for 60 minutes, washed 3x and co-cultured with effector

cells (and human monoclonal antibodies for antibody induced cellular cytotoxicity) in different effector-to-target ratios for 4 to 6 hours or overnight. ^{51}Cr-release in supernatants was assessed on a WIZARD2 gamma-counter (PerkinElmer, Waltham, MA, USA). Specific lysis was calculated as (experimental release-spontaneous release)/(max release-spontaneous release)×100%. Results are expressed as the mean of triplicate samples.

Effector cell responses were assessed by CD107a expression [10]. Beta-cells were incubated with effector cells at different effector target ratios in presence of CD107a-FITC antibody (eBioH4A3, eBiosciences, Vienna, Austria) for 4 hours. Effector cells we counterstained with CD3-PE (UCHT1, BD, Mississauga, Canada), CD8-APC (RPA-T8, BD) for CTLs and CD56-APC (N901, Beckman Coulter, Woerden, The Netherlands) for NK-cells and quantified by flow cytometry (BD FACSCalibur).

For complement dependent cytotoxicity, cells were incubated with antibodies at specified concentrations and incubated 1 hour at room temperature [11]. Thereafter, different concentrations of human serum or rabbit complement (Inno-train, Frankfurt am Main, Germany) were added for another 1 hour. Cell lysis was assessed by propidium iodide staining and measured by flow cytometry (BD FACSCalibur).

To asses apoptosis induction, beta-cell line culture medium was replaced by Th1-cell supernatant or supplemented with 50 U/ml IL-1β (Gentaur, Brussels, Belgium), 1000 U/ml IFNγ (R&D systems) and 1000 U/ml TNF (Gentaur) and cell were cultured for another 48 hours. After harvesting, dead beta cells were assessed by staining with 1 μg/ml propidium iodide on BD FACSCalibur or LSR-II flow cytometers.

Cell surface staining

Cell surface antigens were analysed by flow cytometry on a BD FACSCalibur after 20 minutes staining at 4°C with antibodies to HLA-class I (W6/32; FITC labeled, BD biosciences or biotinilated, local), steptavidin-APC (eBiosciences), HLA-DR-APC (LN3, eBiosciences), HLA-DQ (SPV-L3, *in house,*

labeled with Alexa Fluor 647 kit, Life Technologies), IgG1-APC (MOPC-21, BD), IgG2a-FITC (G155-178, BD), IgG2a-PE (G155-178, BD), anti-CD46-APC (MEM-258, ImmunoTools, Friesoythe, Germany), anti-CD55-PE (IA10, BD), anti-CD59-APC (OV9A2, eBiosciences).

Reference List

[1] Ravassard P, Hazhouz Y, Pechberty S, et al. (2011) A genetically engineered human pancreatic beta cell line exhibiting glucose-inducible insulin secretion. The Journal of clinical investigation 121: 3589-3597

[2] Pahl JH, Ruslan SE, Buddingh EP, et al. (2012) Anti-EGFR antibody cetuximab enhances the cytolytic activity of natural killer cells toward osteosarcoma. Clinical cancer research : an official journal of the American Association for Cancer Research 18: 432-441

[3] Skowera A, Ellis RJ, Varela-Calvino R, et al. (2008) CTLs are targeted to kill beta cells in patients with type 1 diabetes through recognition of a glucose-regulated preproinsulin epitope. The Journal of clinical investigation 118: 3390-3402

[4] Borst J, de Vries E, Spits H, de Vries JE, Boylston AW, Matthews EA (1987) Complexity of T cell receptor recognition sites for defined alloantigens. Journal of immunology 139: 1952-1959

[5] van der Pol P, Roos A, Berger SP, Daha MR, van Kooten C (2011) Natural IgM antibodies are involved in the activation of complement by hypoxic human tubular cells. American journal of physiology Renal physiology 300: F932-940

[6] Nauta AJ, Westerhuis G, Kruisselbrink AB, Lurvink EG, Willemze R, Fibbe WE (2006) Donor-derived mesenchymal stem cells are immunogenic in an allogeneic host and stimulate donor graft rejection in a nonmyeloablative setting. Blood 108: 2114-2120

[7] Roep BO, Arden SD, De Vries RR, Hutton JC (1990) T-cell clones from a type-1 diabetes patient respond to insulin secretory granule proteins. Nature 345: 632-634

[8] Mulder A, Kardol M, Blom J, Jolley WB, Melief CJ, Bruning JW (1993) Characterization of two human monoclonal antibodies reactive with HLA-B12 and HLA-B60, respectively, raised by in vitro secondary immunization of peripheral blood lymphocytes. Human immunology 36: 186-192

[9] Carlotti F, Bazuine M, Kekarainen T, et al. (2004) Lentiviral vectors efficiently transduce quiescent mature 3T3-L1 adipocytes. Molecular therapy : the journal of the American Society of Gene Therapy 9: 209-217

[10] Alter G, Malenfant JM, Altfeld M (2004) CD107a as a functional marker for the identification of natural killer cell activity. Journal of immunological methods 294: 15-22

[11] Pena JR, Fitzpatrick D, Saidman SL (2013) Complement-dependent cytotoxicity crossmatch. Methods in molecular biology 1034: 257-283
